# The value of oral selective estrogen receptor degraders in patients with HR-positive, HER2-negative advanced breast cancer after progression on ≥ 1 line of endocrine therapy: systematic review and meta-analysis

**DOI:** 10.1186/s12885-023-11722-4

**Published:** 2024-01-02

**Authors:** Xiewei Huang, Yushuai Yu, Shiping Luo, Wenfen Fu, Jie Zhang, Chuangui Song

**Affiliations:** 1 Department of Breast Surgery, Fujian Medical University Union Hospital, No. 29, Xin Quan Road, Gulou District, Fuzhou, 350001 Fujian Province China; 2 Department of General Surgery, Fujian Medical University Union Hospital, Fuzhou, 350001 Fujian Province China; 3 Breast Surgery Institute, Fujian Medical University, Fuzhou, 350001 Fujian Province China

**Keywords:** Breast cancer, Advance, HR+/HER2-, Oral SERDs, Meta-analysis

## Abstract

**Background:**

Currently, the value of oral selective estrogen receptor degraders (SERDs) for hormone receptor-positive (HR+) and human epidermal growth factor receptor 2-negative (HER2-) advanced breast cancer (aBC) after progression on ≥ 1 line of endocrine therapy (ET) remains controversial. We conducted a meta-analysis to evaluate progression-free survival (PFS) and safety benefits in several clinical trials.

**Materials and methods:**

Cochrane Library, Embase, PubMed, and conference proceedings (SABCS, ASCO, ESMO, and ESMO Breast) were searched systematically and comprehensively. Random effects models or fixed effects models were used to assess pooled hazard ratios (HRs) and 95% confidence intervals (CIs) for treatment with oral SERDs versus standard of care.

**Results:**

A total of four studies involving 1,290 patients were included in our analysis. The hazard ratio (HR) of PFS showed that the oral SERD regimen was better than standard of care in patients with HR+/HER2- aBC after progression on ≥ 1 line of ET (HR: 0.75, 95% CI: 0.62-0.91, *p* = 0.004). In patients with ESR1 mutations, the oral SERD regimen provided better PFS than standard of care (HR: 0.58, 95% CI: 0.47-0.71, *p <* 0.00001). Regarding patients with disease progression following previous use of CDK4/6 inhibitors, PFS benefit was observed in oral SERD-treatment arms compared to standard of care (HR: 0.75, 95% CI: 0.64-0.87, *p* = 0.0002).

**Conclusions:**

The oral SERD regimen provides a significant PFS benefit compared to standard-of-care ET in patients with HR+/HER2- aBC after progression on ≥ 1 line of ET. In particular, we recommend oral SERDs as a preferred choice for those patients with ESR1m, and it could be a potential replacement for fulvestrant. The oral SERD regimen is also beneficial after progression on CDK4/6 inhibitors combined with endocrine therapy.

**Supplementary Information:**

The online version contains supplementary material available at 10.1186/s12885-023-11722-4.

## Introduction

In the United States, approximately 60-70% of women with advanced breast cancer (aBC) are hormone receptor-positive (HR+) and human epidermal growth factor receptor 2-negative (HER2-) [1–3]. Resistance to treatment, acquisition of novel mutations, and altered gene expression are the major challenges in the management of aBC [4, 5]. There are established guidelines for first-line treatment of these patients, but a consensus has not yet been reached regarding the choice of second-line treatment [6].

Endocrine therapy (ET), with either fulvestrant (Fulv) or aromatase inhibitors (AIs), plus a cyclin-dependent kinase 4/6 inhibitor (CDK4/6i) is the recommended first-line standard of care for patients with HR+/HER2- advanced breast cancer [7]. Compared with endocrine monotherapy, the combination can obtain a higher response rate and progression-free survival benefit [8–10]. However, the development of resistance to the treatment of aBC is frequent, and its treatment is primarily palliative [11] In general, there are three main strategies after the failure of CDK4/6i treatment: diversion to chemotherapy, endocrine therapy alone, or combined targeted therapy [12–14]. Currently, there are no recommended guidelines for the optimal ranking of these options. In any case, ET is still an important treatment strategy.

Estrogen receptor 1 mutations (ESR1m) are one of the common mechanisms of endocrine resistance, accounting for up to 36% of metastatic breast cancers [15, 16]. Selective estrogen receptor degraders (SERDs) can bind to estrogen receptors and induce their degradation [17, 18] and are considered one of the main ways to address endocrine resistance. Fulvestrant, as an intramuscular SERD, is not only the first-line or second-line treatment option for HR+/HER2- aBC [19, 20] but is also a choice for patients with ESR1m, who are still sensitive to it [15, 21, 22]. In recent years, oral SERDs, with their higher bioavailability and pharmacokinetics, have been continuously developed to address the limitations of fulvestrant intramuscular formulations [23]. However, the value of oral SERDs in patients with HR+/HER2- advanced breast cancer remains controversial. EMERALD [24] and SERENA-2 [25] showed positive results, while the other two clinical trials, AMEERA-3 [26] and acelERA [27], failed the study endpoints.

In the present meta-analysis, we aimed to assess the value of oral SERDs in patients with HR+/HER2- advanced breast cancer after progression on ≥ 1 line of endocrine therapy.

## Materials and methods

### Search strategy and data extraction

The systematic review of literature and meta-analysis was conducted according to the Preferred Reporting Items for Systematic Reviews and Meta-Analyses (PRISMA) guidelines [28]. The corresponding PRISMA checklist is shown in Supplement 2. A systematic and comprehensive literature search was conducted using Embase, PubMed, and Cochrane Library**.** Conference proceedings from major oncology meetings (ASCO, SABCS, ESMO, and ESMO Breast) from 2020 up to November 2023 were also carefully reviewed. The following search string was used: “(breast OR mammary) AND (cancer OR carcinoma OR malignant OR neoplasm OR tumour) AND (hormone receptor-positive OR HR-positive OR HR OR estrogen receptor-positive OR ER OR ER-positive) AND (HER-2- OR HER2- OR ERBB2- OR HER-2 negative OR HER2-negative OR ERBB2 negative OR human epidermal growth factor receptor 2-negative) AND (metastasis OR metastases OR metastatic OR advanced OR recurrent OR stage IV) AND (oral selective estrogen receptor degrader OR SERD OR Giredestrant OR Camizestrant OR Imlunestrant OR Elacestrant OR Amcenestrant).” Records from the included studies were screened independently by two investigators. In cases of disagreement, the third investigator was consulted to reach a consensus.

Details about the title, publication date, study design, and trial name were extracted. All relevant randomized controlled trials were identified as the recommendations of the Cochrane Collaboration [29]. When duplicate publications were identified, only the latest data were extracted in our study. Other details about the first author, country, sample size, menopausal status, oral SERDs used, dose of oral SERDs, treatment regimens used in the control arm, previous treatment regimen, ESR1m status, hazard ratio (HR), progression-free survival (PFS), median progression-free survival (mPFS) and side effects for each arm were extracted. The primary outcome was progression-free survival, which was defined as the time from randomization to death or disease progression, whichever occurred first. The proportion of patients who achieved an overall response according to the Response Evaluation Criteria in Solid Tumours (RECIST) was selected as a secondary outcome [30]. An exploratory analysis was conducted based on the Common Terminology Criteria for Adverse Events, version 4, reporting the proportion of patients with grade 3-5 adverse events [31]. All data included in the study were extracted independently by two investigators.

### Study selection

Studies had to satisfy the following inclusion and exclusion criteria: (I) phase II or III randomized clinical trials (RCTs) including patients with HR+/HER2- aBC after progression on ≥ 1 line of ET; (II) comparison of oral SERD-treated patients and patients treated with standard-of-care ET; and (III) the publication provided PFS and HR for the experimental and control arms. Systemic reviews, case reports, single-arm studies, exploratory studies, and retrospective studies were excluded. If multiple publications were associated with the same clinical trial, only the latest and complete randomized controlled trial was included.

### Objectives

The primary objective of the study was to compare the efficacy of oral SERDs with standard-of-care ET in patients with HR+/HER2- aBC after progression on ≥ 1 line of ET. The secondary objective was to analyse the subgroup of patients in the population that might benefit from oral SERDs. We planned the subgroup analysis for the following subgroups: patients with disease progression following previous use of CDK4/6 inhibitors or Fulv; patients with ESR1m; patients with visceral metastasis; comparing oral SERDs with fulvestrant; and comparing oral SERDs with fulvestrant in patients with ESR1m.

### Statistical analysis

Global PFS was calculated using a random-effects model or fixed-effects model and reported as pooled hazard ratios (HRS) with 95% confidence intervals (CIs). If the 95% CI did not include 1.0 and the two-sided threshold was *P* < 0.05, the pooled HR was considered statistically significant. The I^2^ value was employed for the heterogeneity of included studies. When I^2^ > 50%, significant heterogeneity was considered established, and the random-effects model was adopted; otherwise, the fixed-effects model was used. When heterogeneity was high in the pooled results, sensitivity analysis was performed after every single study was excluded. All statistical analysis methods were performed using Review Manager (version 5.3). The Cochrane Collaboration’s Risk of Bias tool in Review Manager (version 5.3) was employed to assess the risk of bias for each eligible study.

## Results

### Study selection

**Fig. 1:**
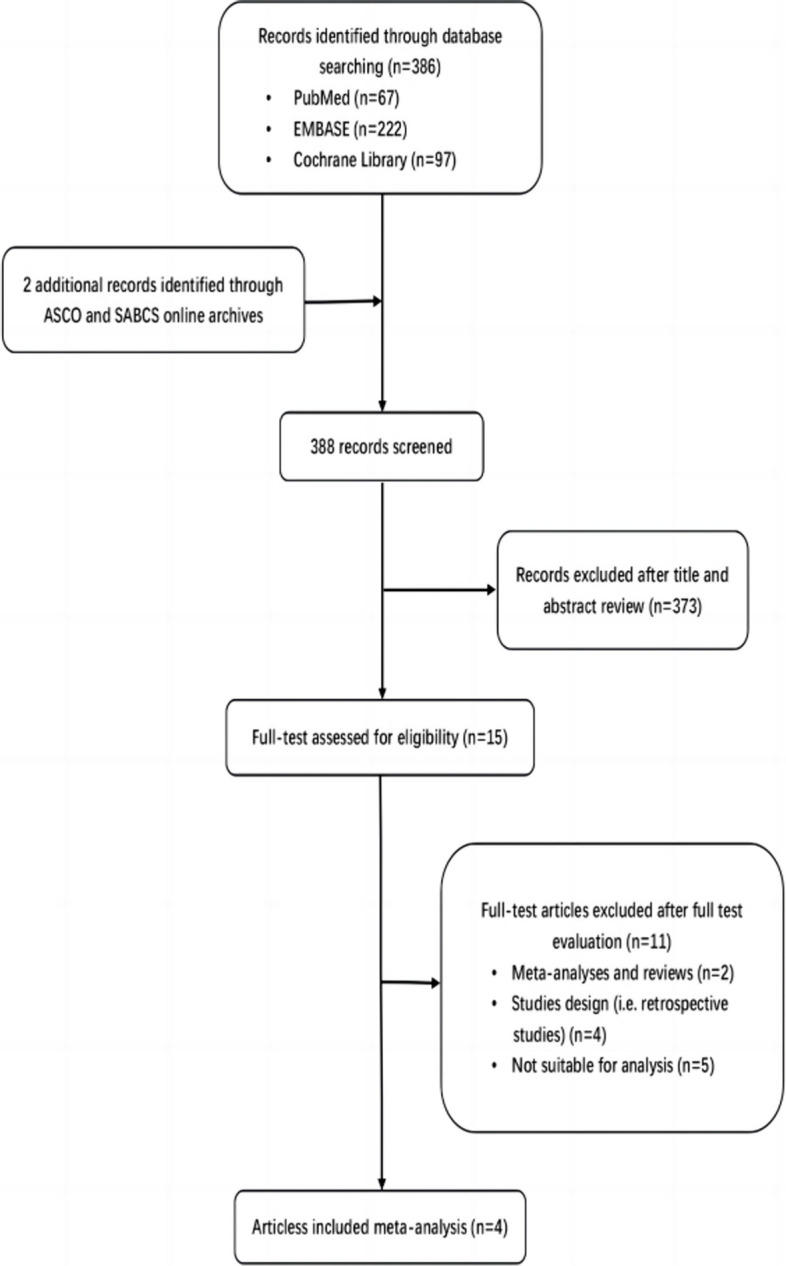
PRISMA flowchart for the selected studies included in the meta-analysis

A total of 386 potentially relevant manuscripts and 2 additional abstracts were sorted by using the search string mentioned before. Of these, after reviewing the titles and abstracts, 373 manuscripts were excluded. We then performed a full-text review for the remaining 15 articles, 11 of which were excluded for nonconformity with the present inclusion criteria. Eventually, 4 articles from 4 trials were considered eligible for the meta-analysis. The Preferred Reporting Items for Systematic Reviews and Meta-Analysis (PRISMA) flowchart is shown in Fig. 1.

### Characteristics of studies

Finally, our study involved 4 clinical trials published between February 2022 and November 2023, focusing on different endocrine treatment regimens for HR+/HER2- advanced breast cancer, and included a total of 1,290 patients (Table 1). The oral SERD arms included elacestrant (EMERALD), camizestrant 75 mg/camizestrant 150 mg (SERENA-2), amcenestrant (AMEERA-3), and giredestrant (acelELA). The control arms included fulvestrant, anastrozole, letrozole, exemestane, and tamoxifen. All trials compared oral SERDs to standard-of-care ET in patients with HR+/HER2- aBC after progression on ≥ 1 line of ET.

**Table 1:** Characteristics of eligible studies in the meta-analysis

Study	EMERALD	SERENA-2	AMEERA-3	acelELA
First author	Francois-Clement Bidard	Mafalda Oliveira	Sara M. Tolaney	Miguel Martin
Year of publication	2022	2022	2023	2022
Phase	III	II	II	II
Patients, n	477	220	290	303
Patients	Men or postmenopausal women	Postmenopausal women	Men or women (any menopausal status)	Men or women (any menopausal status)
Oral SERD regimen/dose	Elacestrant/400 mg	Camizestrant/75 mg (A)^a^/ 150 mg (B)^a^	Amcenestrant/400 mg	Giredestrant/30 mg
Standard-of-care ET	SOC	Fulvestrant	TPC	PCET
ESR1m, n	228	68	120	90
Prior CDK4/6i, %	Required, 100	Permitted, 49.6	Permitted, 79	Permitted, 42
Allowed prior fulvestrant	Yes	No	Yes	Yes
HR	0.70	0.58 (75 mg)/0.67 (150 mg)	1.051	0.81
95% CI	0.55-0.88	0.41-0.81 (75 mg)/0.48-0.92 (150 mg)	0.789-1.40	0.60-1.01

*Abbreviations*: *SOC* Standard-of-care, *TPC* Treatment of physician’s choice, *PCET* Physician’s choice of endocrine monotherapy

^a^SERENA-2 was divided into two cohorts because the comparisons were between two doses of camizestrant 75 mg and 150 mg

### Progression-free survival

**Fig. 2:**
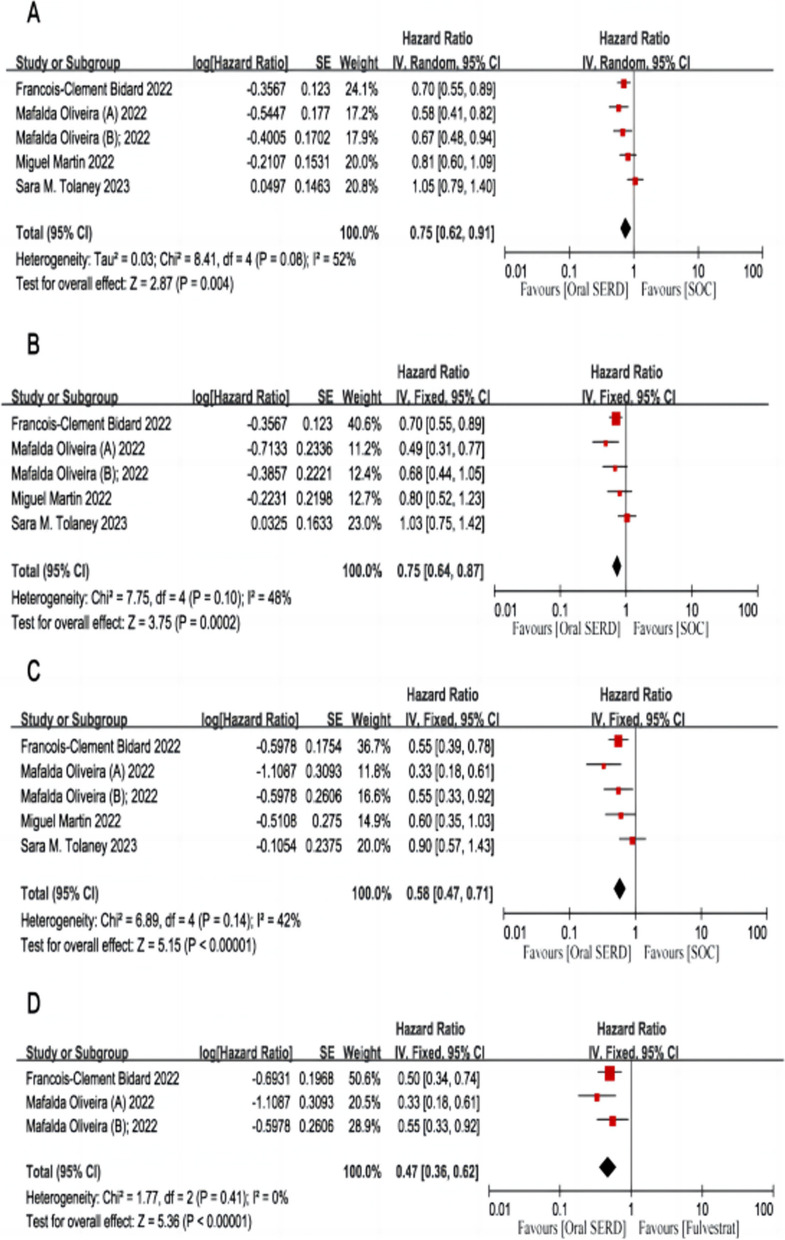
The Forrest plot of PFS for patients with HR+/HER2- advanced breast cancer after progression on ≥ 1 line of endocrine treatment. **A** PFS pooled result for overall patients; **B** PFS pooled result for patients with previous use of CDK4/6 inhibitors; **C** PFS pooled result for patients with ESR1m; **D** PFS pooled result for comparing oral SERDS with fulvestrant in patients with ESR1m subgroup. Note: PFS, progression-free survival; CI, confidence interval; HR, hazard ratio; HR+/HER2-, hormone receptor-positive and human epidermal growth factor receptor 2-negative; SERDs, selective estrogen receptor degraders; ESR1m, estrogen receptor 1 mutations

**Fig. 3:**
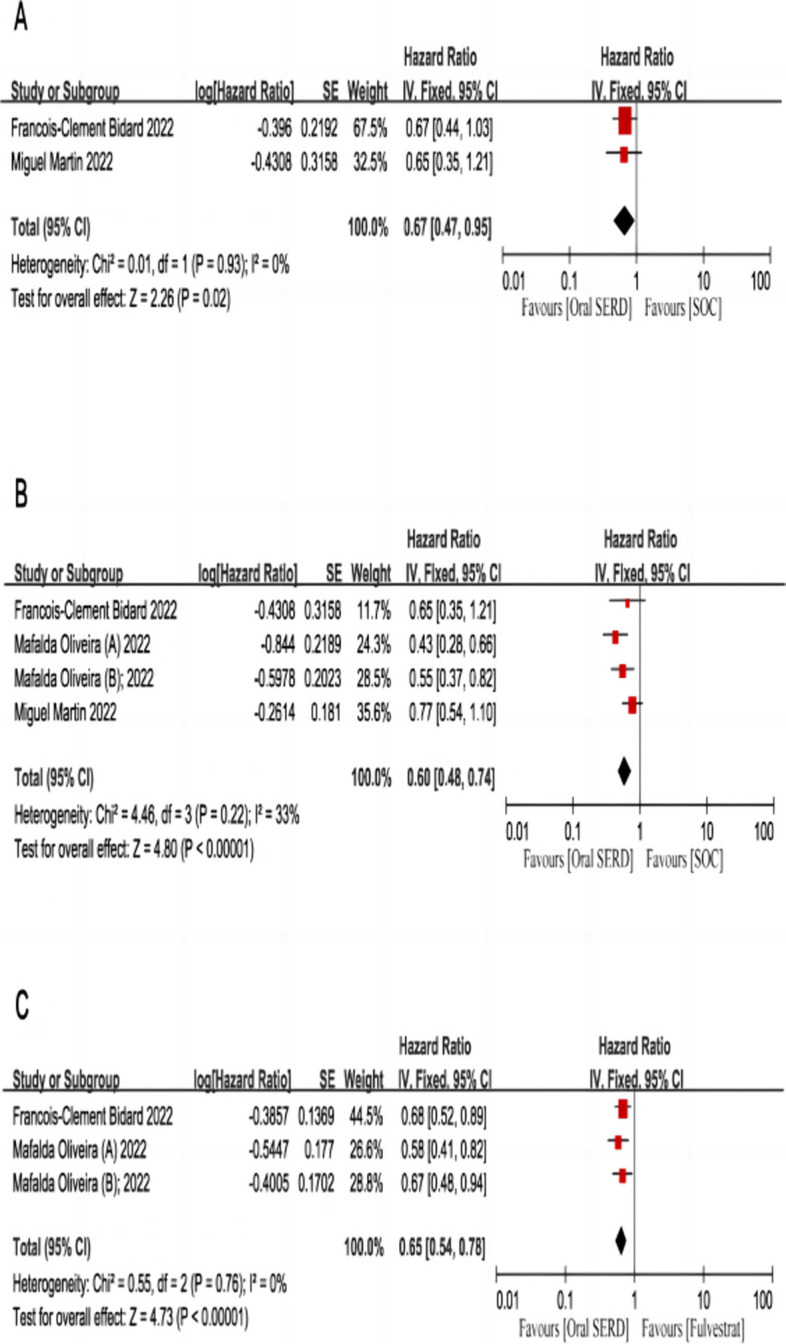
The Forrest plot for global PFS for patients with (**A**) previous use of fulvestrant; (**B**) visceral metastasis; (**C**) Forrest plot for global PFS comparing oral SERDS with fulvestrant. Note: PFS, progression-free survival; CI, confidence interval; HR, hazard ratio; HR+/HER2-, hormone receptor-positive and human epidermal growth factor receptor 2-negative; SERDs, selective oestrogen receptor degraders

In the whole population, patients with HR+/HER2- advanced breast cancer treated with oral SERDs had significantly improved PFS compared to those treated with standard-of-care ET (HR: 0.75, 95% CI: 0.62-0.91, *p* = 0.004; I^2^: 52%, *p* = 0.08; Fig. 2A). For enrolled patients with disease progression following previous use of CDK4/6 inhibitors, the oral SERD regimen was significantly better than standard-of-care ET (HR: 0.75, 95% CI: 0.64-0.87, *p* = 0.0002; I^2^: 48%, *p* = 0.10; Fig. 2B). In HR+/HER2- ESR1m aBC, the two treatment regimens compared, namely, oral SERDs resulted in a better PFS versus standard-of-care ET (HR: 0.58, 95% CI: 0.47-0.71, *p* < 0.00001; I^2^: 42%, *p =* 0.14; Fig. 2C). Regarding enrolled patients with ESR1 mutations, results in arms of oral SERDs were significantly better than in arms of fulvestrant (HR: 0.47, 95% CI: 0.36-0.62, *p* < 0.00001; I^2^: 0%, *p* = 0.41; Fig. 2D). Regarding patients who had previously failed treatment with fulvestrant, oral SERDs as monotherapy were significantly superior to standard-of-care ET (HR: 0.67, 95% CI: 0.47-0.95, *p* = 0.02; I^2^: 0%, *p* = 0.93; Fig. 3A). In patients with visceral disease, the results in arms of oral SERDs were significantly better than the results in arms of standard-of-care ET (HR: 0.60, 95% CI: 0.48-0.74, *p* < 0.00001; I^2^: 33%, *p* = 0.22; Fig. 3B). The results in arms of oral SERDs were significantly better than those in arms of fulvestrant (HR: 0.65, 95% CI: 0.54-0.78, *p* < 0.00001; I^2^: 0%, *p* = 0.76; Fig. 3C).

### Safety

**Fig. 4:**
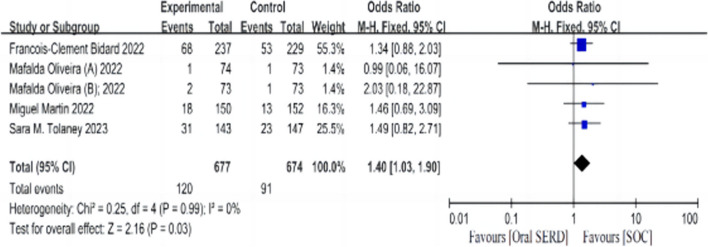
The Forrest plot for AE ≥ Grade 3 for patients with HR+/HER2- advanced breast cancer after progression on ≥ 1 line of ET. Note: AE, adverse event; progression-free survival; CI, confidence interval; HR, hazard ratio; HR+/HER2-, hormone receptor-positive and human epidermal growth factor receptor 2-negative; SERDs, selective estrogen receptor degrader

Adverse events (AEs) of grade 3 or higher were more frequent in the oral SERD regimen than in standard-of-care ET (HR: 1.40, 95% CI: 1.03-1.90, *p* = 0.03; I^2^: 0%, *p* = 0.99; Fig. 4). The proportion of treatment-emergent adverse events (TEAEs) leading to discontinuation was 6.3% (Elacestrant) vs. 4.4% (SOC) in EMERALD's two treatment arms. The most common adverse event was nausea. The proportion of drug discontinuation caused by treatment related AEs (TRAEs) in the three treatment groups of SERENA-2 was 2.7% (camizestrant 75 mg), 0% (camizestrant 150 mg), and 0% (fulvestrant as standard-of-care ET), respectively; common adverse events were photopsia and sinus bradycardia. In AMEERA-3, the proportion of TRAEs ≥ Grade 3 was 4.9% in the experimental arm and 0.7% in the control arm. The most common adverse event was nausea. In acelELA, the incidence of AE ≥ Grade 3 was 12% (giredestrant) vs. 8.6% (physician’s choice of endocrine monotherapy); the most common adverse event was hepatotoxicity.

### Bias assessment

In all trials included, the overall risk of bias was low (Supplement 1 Fig. 1). Since these trials were conducted with an open-label design, performance bias that did not affect the results may exist. There was no obvious publication bias (Supplement 1 Figs. 2 and 3).

## Discussion

Our study showed that the oral SERD regimen was superior to standard-of-care ET in patients with HR+/HER2- advanced breast cancer after progression on ≥ 1 line of ET. However, the characteristics of these patients were complex, so it is crucial to select the characteristics of those patients who are likely to have sustained benefits.

Patients with ESR1m develop resistance to ET and exhibit worse overall survival [32–34]. Our meta-analysis showed that for patients with ESR1 mutations, outcomes in the arms of oral SERDs were significantly better than those in the arms of standard-of-care ET. Surprisingly, in these four clinical trials, oral SERDs were able to provide PFS benefits in ESR1m patients. In addition, patients with ESR1m showed a trend of OS improvement in Elacestrant (HR = 0.59; *p* = 0.03). AIs not only enhance the acquisition of ESR1 mutations in aBC, but patients with ESR1 mutations also showed a worse prognosis in AI treatment [35]. However, patients with ESR1 mutations remained sensitive to fulvestrant [15, 21, 22]. As an intramuscular SERD, fulvestrant binds to estrogen receptors and induces their degradation, [17, 18] so it still plays a role in patients with ESR1 mutations. A pooled analysis of patients with ESR1 mutations in the EFECT and SoFEA trials (115/383) found no significant difference in PFS in the Fulv group (3.9 months versus 4.1 months) [36–38]. However, the clinical utilization of Fulv is limited by its intramuscular formation. In the Elacestrant and SERENA-2 trials, the arms of oral SERDs were significantly better than the arms of fulvestrant (HR: 0.47, p < 0.00001). In addition, its better bioavailability and patient preference for oral medication may lead to better compliance. Patient tolerability of the drug also needs to be considered. The overall toxicity of oral SERDs was found to be greater in our analysis. However, considering that a proportion of patients in the control arms were on AI and tamoxifen regimens, the toxicity of AIs and tamoxifen was lower than that of Fulv [39–41]. Therefore, this does not mean that oral SERDs are more toxic than Fulv. Moreover, treatment resistance to Fulv leading to disease progression remains a major concern for HR+/HER2- aBC. Therefore, both additional endocrine therapy and effective combination therapy are clinically necessary [15, 16]. Data from the Elacestrant and acelELA trials also support oral SERD regimens for patients who failed Fulv therapy. Thus, oral SERDs are recommended in HR+/HER2- ESR1m aBC after ET ≥ 1 line progression, and oral SERDs could be a potential replacement for Fulv.

For HR+/HER2- aBC patients who progressed after first-line treatment with ET combined with CDK4/6i, the oral SERD regimen also had a statistically significant PFS benefit. In the event of disease progression during the use of CDK4/6is, ET-based regimens remain an appropriate option [12, 13]. Patients' menopausal status, tolerance to drugs, and previous treatment regimens will affect the subsequent selection of endocrine agents [42]. These enrolled patients had previously used one or two ET regimens, so it is still necessary to find new endocrine agents. Camizestrant therapy may be a new option for these patients. The median PFS in the oral SERDs group was 7.2 (75 mg) and 7.7 (150 mg) months, respectively, while that in the Fulv group was only 3.7 months. Even in the subgroup with previous use of CDK4/6i, there was a significant improvement in PFS [median PFS 5.5 (75 mg) and 3.8 (150 mg) months vs. 2.1 months]. However, the absolute benefit in Elacestrant was very small (median PFS 2.8 months vs. 1.9 months). In ESR1m aBC patients previously treated with CDK4/6i for ≥12 months, elacestrant had a median PFS of 8.6 months and SOC of 2.1 months, which was a clinically and statistically significant improvement. This suggests that a possible indication for elacestrant may be the duration of previous CDK4/6i [43]. In addition, in those patients with visceral metastasis, oral SERDs also showed advantages (HR: 0.60, *P* < 0.00001). Endocrine therapy is the preferred option for HR+ breast cancer patients even in the presence of visceral metastases [44]. Compared with endocrine monotherapy, the combination can obtain a higher response rate and progression-free survival benefit [45]. Chemotherapy is recommended for patients with visceral crisis. However, chemotherapy is more toxic and causes many side effects in patients [46]. In contrast, oral SERDs show better efficacy in patients with visceral metastasis and can also reduce the serious side effects caused by chemotherapy.

EMERALD and SERENA-2 showed positive results in these four randomized controlled trials, while the other two trials, AMEERA-3 and acelERA, failed the study endpoints. Due to the heterogeneity of enrolled patients and differences in control settings, indirect cross-comparisons between different trials should be undertaken with caution. First, prior treatment regimens after disease progression varied across the four trials. In the SERENA-2 trial, 31.3% of patients had previously not received ET in the advanced setting, whereas in the other three trials, patients had previously received at least one or two lines of ET. Studies have shown that monotherapy with Fulv had advantages in PFS compared to aromatase inhibitors or tamoxifen monotherapy [47, 48]. In the control arm of AMEERA-3 and acelERA, the proportion of patients treated with Fulv was higher (89.8% and 75%, respectively), which may have resulted in prolonged mPFS in the control group. In addition, all patients in the SERENA-2 control group received Fulv, but previous Fulv was not permitted for aBC patients. In EMERALD, however, 30.4% of patients had previously been treated with Fulv; in AMEERA-3, the corresponding value was 9.7%, and in acelERA, it was 26.19%.

Our study is the first to evaluate the value of oral SERDs in patients with HR+/HER2- aBC after progression on ≥ 1 line of endocrine therapy. The characteristics of the population that may benefit are also analysed. Especially for patients with ESR1m, oral SERDs are advantageous. Further screening of advantaged oral SERD groups for stratified treatment is the future development trend. The value of SERDs may not be limited to patients in advanced settings. Studies such as CAMBRIA-1 [49] are being conducted to assess the potential of oral SERDs in early-stage breast cancer. In addition, oral dosage forms are more convenient. This can save manpower and material resources to a certain extent, and the compliance of patients will be better. It is believed that it will have good application prospects. There are several limitations to our study. First, this was not a network meta-analysis, and we could not directly compare all drugs or drug combinations with each other. As a result, a certain degree of precision was lost. In addition, we could not evaluate the overall survival (OS) benefit due to the unavailability of data. Although OS is the "gold standard" for efficacy evaluation in cancer clinical research, it has certain limitations in practical application. OS as the primary endpoint requires a large sample size, and clinical development is difficult. It is affected by death from nontumour causes. For tumour types with long survival, the duration of the study is extremely long. Therefore, alternative end points are often used for those patients with long survival, and the FDA currently supports the use of PFS as an end point. However, these limitations are unavoidable at present. At present, there are relatively few studies on oral SERDs, and it is hoped that more clinical trials will follow to confirm our experiments.

## Conclusion

The oral SERD regimen has a significant PFS benefit compared to standard-of-care ET in patients with HR+/HER2- aBC after progression on ≥ 1 line of ET. In particular, we recommend oral SERDs as a preferred choice for those patients with ESR1m, and it could be a potential replacement for fulvestrant. The oral SERD regimen also benefits after progression on CDK4/6 inhibitors combined with endocrine therapy.

### Supplementary Information

**Additional file 1:** **Supplementary Figure 1.** Quality assessment for the bias items of RCTs. (a) Risk of the bias summary. (b) Risk of the bias graph. **Supplementary Figure 2.** The funnel plot PFS for patients with HR+/HER2- advanced breast cancer after progression on ≥ 1 line of endocrine treatment: (A) The funnel plot PFS for overall patients; (B) The funnel plot PFS for patients with previous use of CDK4/6 inhibitors; (C) The funnel plot PFS for  patients with ESR1m; (D) The funnel plot PFS for comparing oral SERDS with fulvestrant in patients with ESR1m subgroup. Note: PFS, progression-free survival; CI, confifidence interval; HR, hazard ratio; HR+/HER2-, hormone receptor-positive and human epidermal growth factor receptor 2-negative; SERDS, selective estrogen receptor degrader; ESR1m, estrogen receptor 1 mutations. **Supplementary Figure 3.** The funnel plot PFS for patients with (A) previous use of fulvestrant; (B) visceral metastasis; (C) funnel plot for PFS comparing oral SERDS with fulvestrant. (D) The funnel plot for AE ≥ Grade 3 for patients with HR+/HER2- advanced breast cancer after progression on ≥ 1 line of ET. Note: PFS, progression-free survival; CI, confifidence interval; HR, hazard ratio; HR+/HER2-, hormone receptor-positive and human epidermal growth factor receptor 2-negative; SERDS, selective estrogen receptor degrader; AE, adverse event.

**Additional file 2.** 

## Data Availability

All data generated or analysed during this study are included in this published article [and its supplementary information files].
