# Correction: The value of oral selective estrogen receptor degraders in patients with HR-positive, HER2-negative advanced breast cancer after progression on ≥ 1 line of endocrine therapy: systematic review and meta-analysis

**DOI:** 10.1186/s12885-024-11932-4

**Published:** 2024-02-13

**Authors:** Xiewei Huang, Yushuai Yu, Shiping Luo, Wenfen Fu, Jie Zhang, Chuangui Song

**Affiliations:** 1 Department of Breast Surgery, Fujian Medical University Union Hospital, No. 29, Xin Quan Road, Gulou District, 350001 Fuzhou, Fujian Province China; 2 Department of General Surgery, Fujian Medical University Union Hospital, 350001 Fuzhou, Fujian Province China; 3 Breast Surgery Institute, Fujian Medical University, 350001 Fuzhou, Fujian Province China

**Correction: BMC Cancer 24, 21 (2024)**

10.1186/s12885-023-11722-4

Following publication of the original article [1], the authors reported an error in the fourth sentence of the first paragraph of the “Safety” sub-section of the “Results” section (copied and in bold below for your ease of reference): **The proportion of drug discontinuation caused by TEAEs in the three treatment groups of SERENA-2 was 14.9% (camizestrant 75 mg), 21.9% (camizestrant 150 mg), and 4.1% (standard-of-care ET), respectively.** We have quoted the proportion of TEAEs leading to dose interruption, rather than those leading to discontinuation which is an error. The correct sentence should be: **The proportion of drug discontinuation caused by treatment related AEs (TRAEs) in the three treatment groups of SERENA-2 was 2.7% (camizestrant 75 mg), 0% (camizestrant 150 mg), and 0% (fulvestrant as standard-of-care ET), respectively.**

The original article [1] has been corrected.
