# Supplementary material for: The value of oral selective estrogen receptor degraders in patients with HR-positive, HER2-negative advanced breast cancer after progression on ≥ 1 line of endocrine therapy: systematic review and meta-analysis
Source: BMC Cancer. 2024 Jan 2;24:21. doi: 10.1186/s12885-023-11722-4 (PMC10763362; doi:10.1186/s12885-023-11722-4)
Supplement: Supplementary file 1 — Additional file 1: Supplementary Figure 1. Quality assessment for the bias items of RCTs. (a) Risk of the bias summary. (b) Risk of the bias graph. Supplementary Figure 2. The funnel plot PFS for patients with HR+/HER2- advanced breast cancer after progression on ≥ 1 line of endocrine treatment: (A) The funnel plot PFS for overall patients; (B) The funnel plot PFS for patients with previous use of CDK4/6 inhibitors; (C) The funnel plot PFS for patients with ESR1m; (D) The funnel plot PFS for comparing oral SERDS with fulvestrant in patients with ESR1m subgroup. Note: PFS, progression-free survival; CI, confifidence interval; HR, hazard ratio; HR+/HER2-, hormone receptor-positive and human epidermal growth factor receptor 2-negative; SERDS, selective estrogen receptor degrader; ESR1m, estrogen receptor 1 mutations. Supplementary Figure 3. The funnel plot PFS for patients with (A) previous use of fulvestrant; (B) visceral metastasis; (C) funnel plot for PFS comparing oral SERDS with fulvestrant. (D) The funnel plot for AE ≥ Grade 3 for patients with HR+/HER2- advanced breast cancer after progression on ≥ 1 line of ET. Note: PFS, progression-free survival; CI, confifidence interval; HR, hazard ratio; HR+/HER2-, hormone receptor-positive and human epidermal growth factor receptor 2-negative; SERDS, selective estrogen receptor degrader; AE, adverse event. [file 12885_2023_11722_MOESM1_ESM.docx]

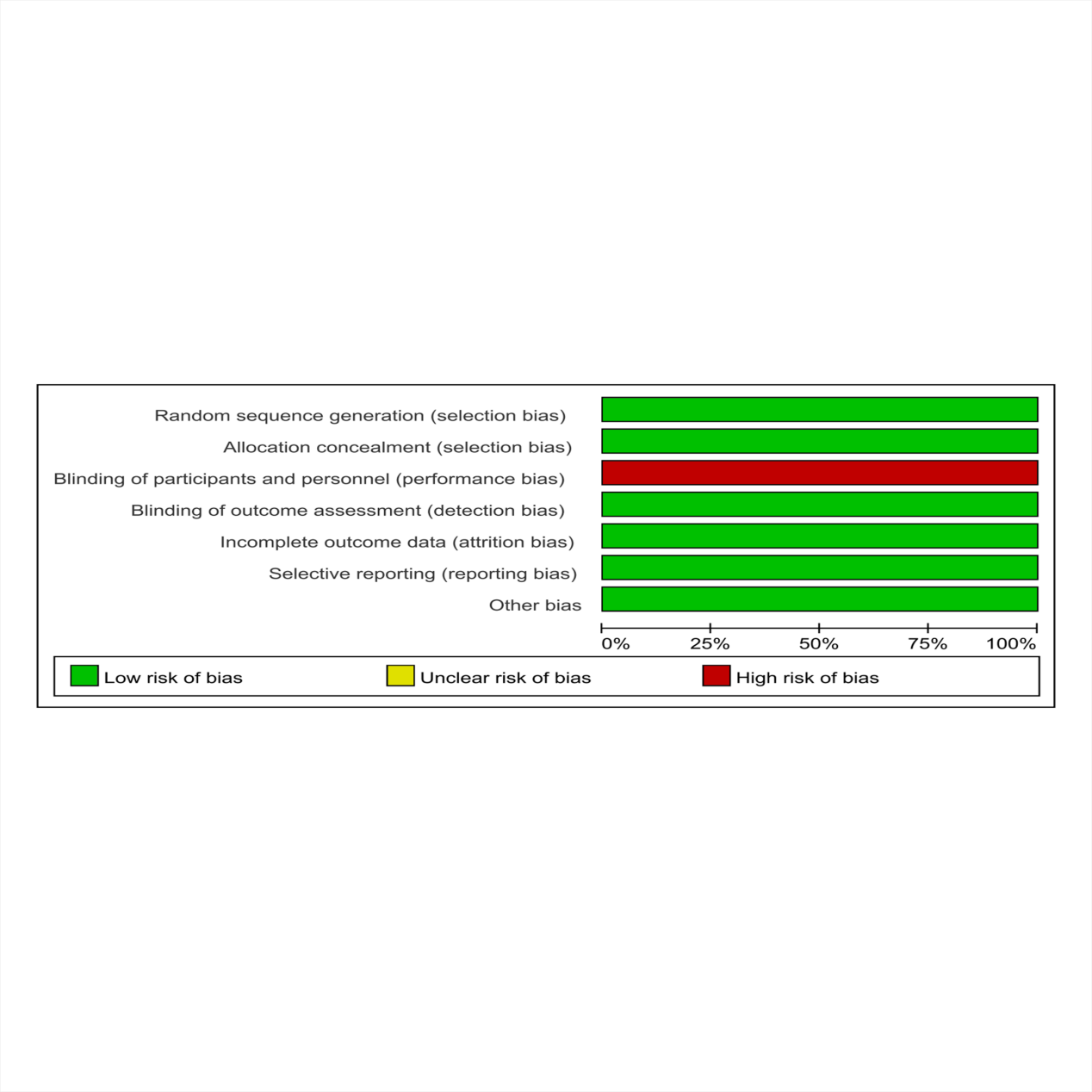
(a)


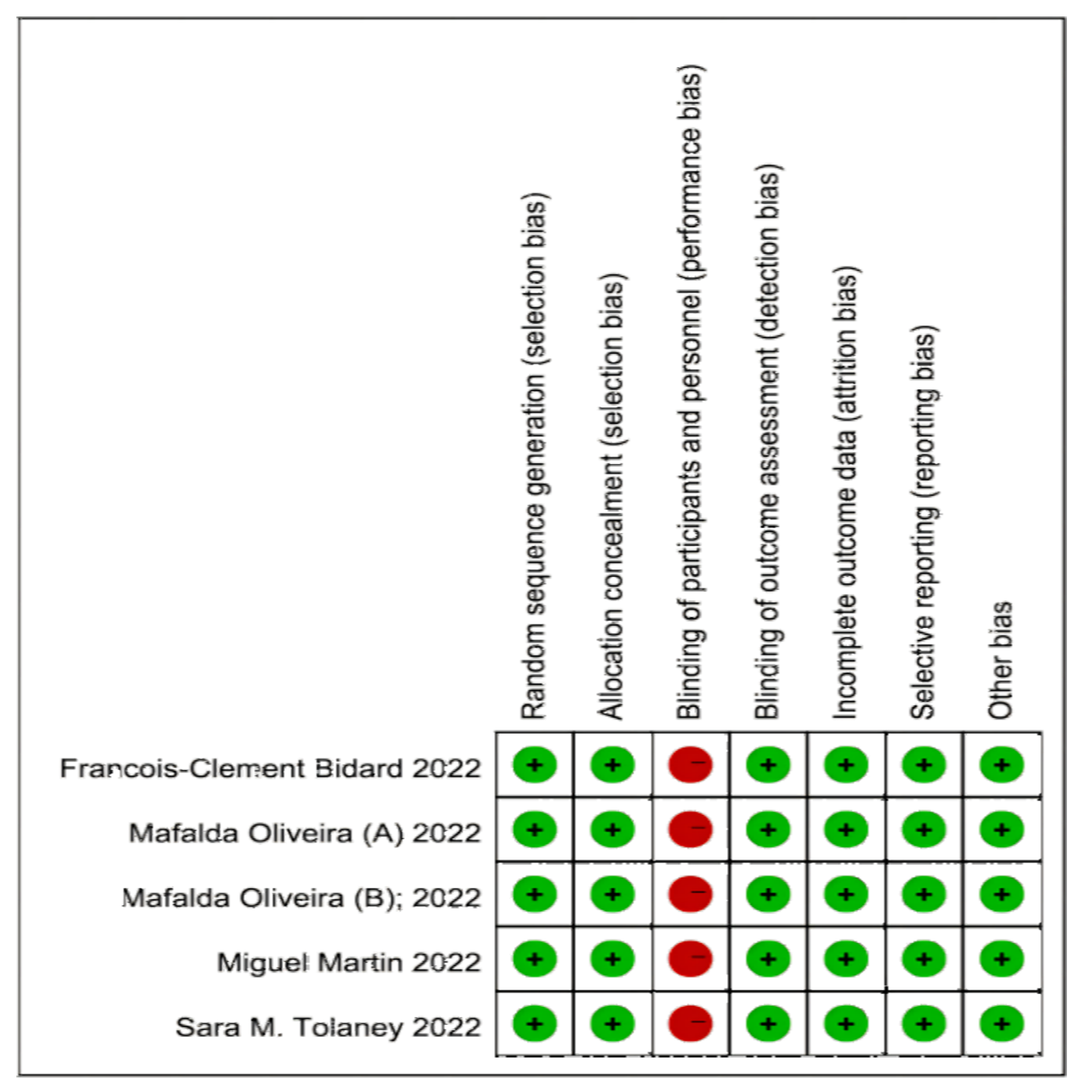


(b)

**Supplementary Figure 1.** Quality assessment for the bias items of RCTs. (a) Risk of the bias summary. (b) Risk of the bias graph.

**
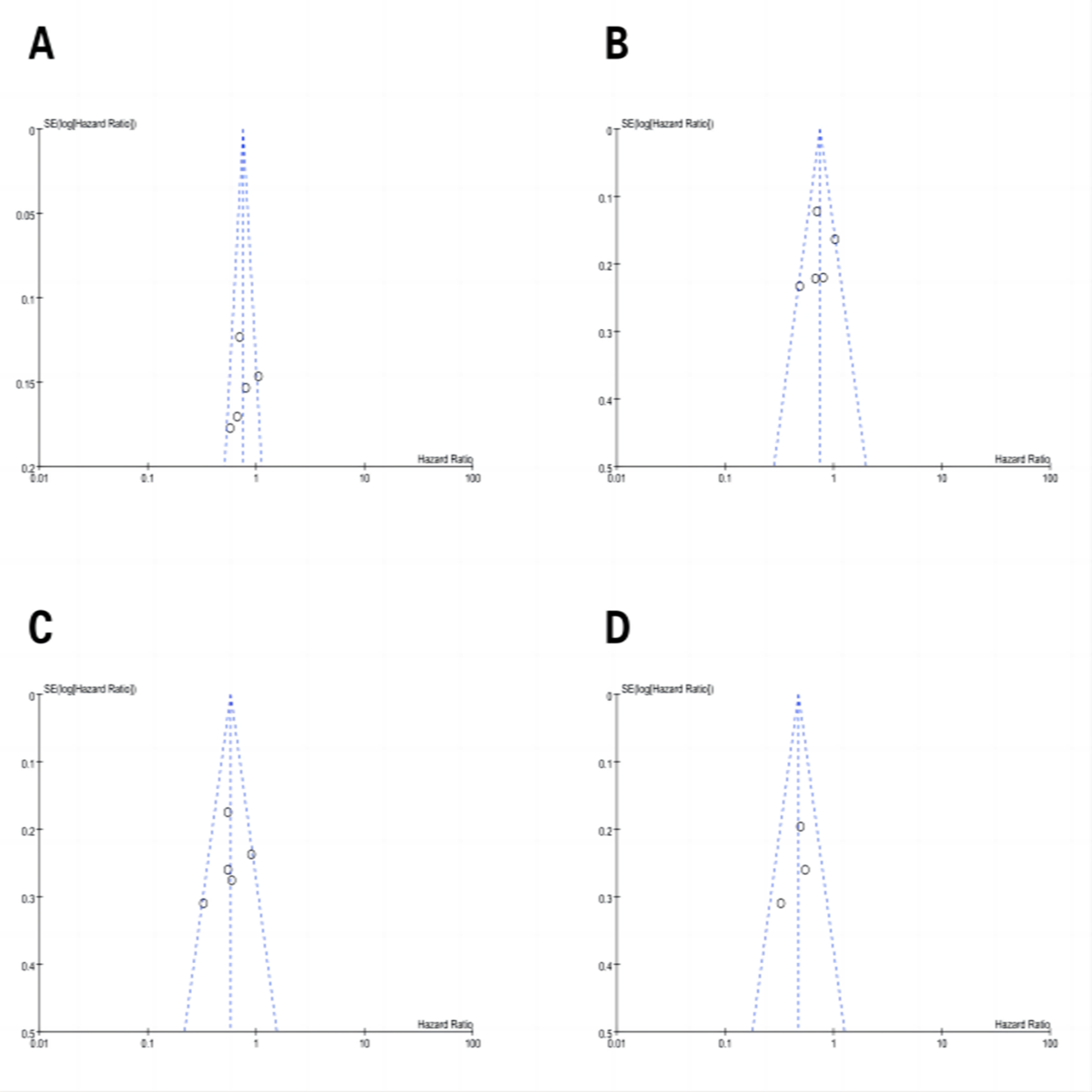
**

**Supplementary Figure 2.** The funnel plot PFS for patients with HR+/HER2- advanced breast cancer after progression on ≥ 1 line of endocrine treatment: (A) The funnel plot PFS for overall patients; (B) The funnel plot PFS for patients with previous use of CDK4/6 inhibitors; (C) The funnel plot PFS for patients with ESR1m; (D) The funnel plot PFS for comparing oral SERDS with fulvestrant in patients with ESR1m subgroup. Note: PFS, progression-free survival; CI, confifidence interval; HR, hazard ratio; HR+/HER2-, hormone receptor-positive and human epidermal growth factor receptor 2-negative; SERDS, selective estrogen receptor degrader; ESR1m, estrogen receptor 1 mutations.


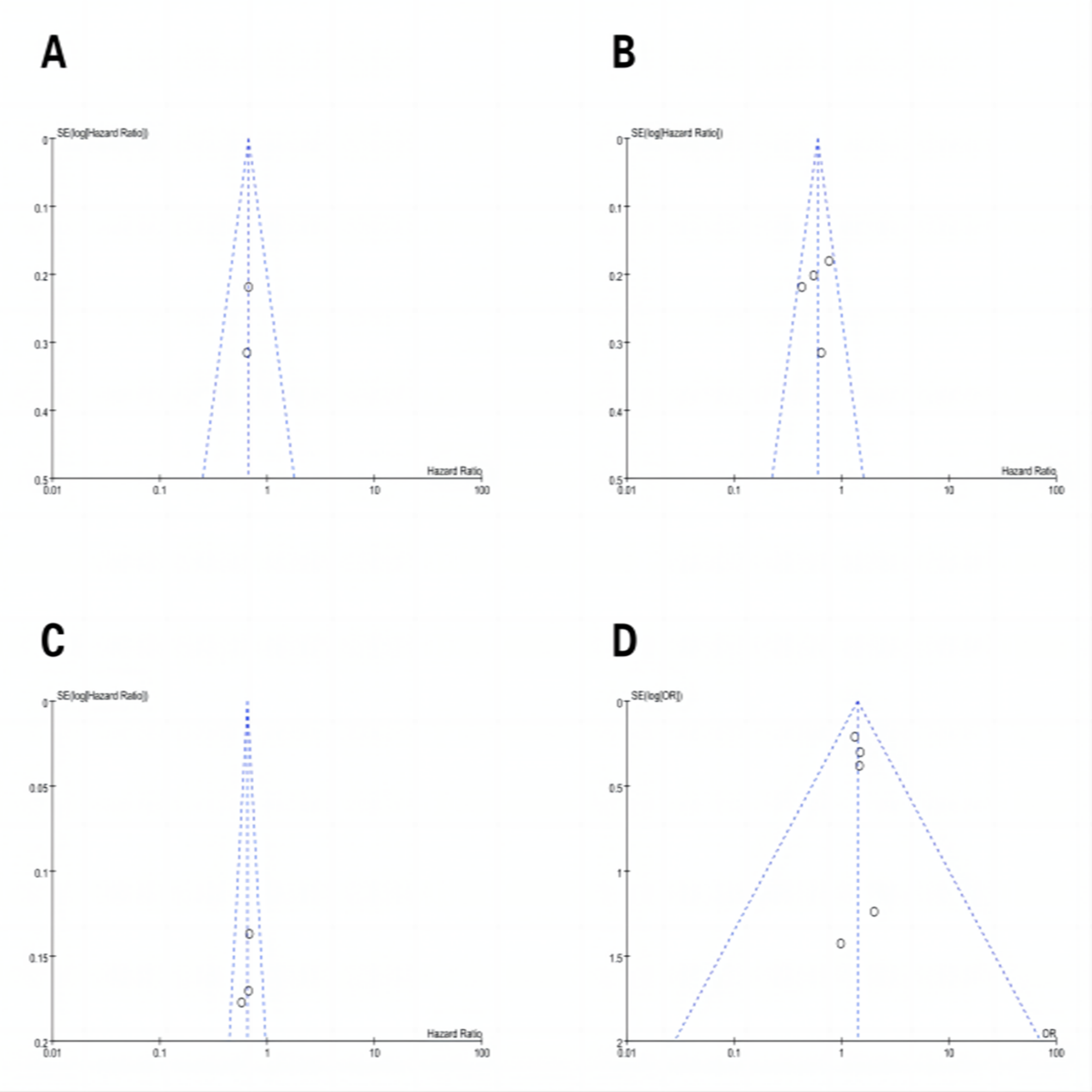


**Supplementary Figure 3.** The funnel plot PFS for patients with (A) previous use of fulvestrant; (B) visceral metastasis; (C) funnel plot for PFS comparing oral SERDS with fulvestrant. (D) The funnel plot for AE ≥ Grade 3 for patients with HR+/HER2- advanced breast cancer after progression on ≥ 1 line of ET.

Note: PFS, progression-free survival; CI, confifidence interval; HR, hazard ratio; HR+/HER2-, hormone receptor-positive and human epidermal growth factor receptor 2-negative; SERDS, selective estrogen receptor degrader; AE, adverse event.
